# Point-specific interactions of isovitexin with the neighboring amino acid residues of the hACE2 receptor as a targeted therapeutic agent in suppressing the SARS-CoV-2 influx mechanism

**DOI:** 10.5455/javar.2022.i588

**Published:** 2022-06-26

**Authors:** Nourin Ferdausi, Samarth Islam, Fahmida Hoque Rimti, Syeda Tasnim Quayum, Efat Muhammad Arshad, Aashian Ibnat, Tamnia Islam, Adittya Arefin, Tanzila Ismail Ema, Partha Biswas, Dipta Dey, Salauddin Al Azad

**Affiliations:** 1 Biochemistry and Molecular Biology, University of Dhaka, Dhaka, Bangladesh; 2 Department of Biochemistry and Microbiology, North South University, Dhaka, Bangladesh; 3 Bachelor of Medicine and Surgery, Chittagong Medical College, Chittagong, Bangladesh; 4 Wolfson Institute for Biomedical Research, Division of Medicine, University College London, London, United Kingdom; 5 Department of Pharmaceutical Sciences, North South University, Dhaka, Bangladesh; 6 Department of Genetic Engineering and Biotechnology, Jashore University of Science and Technology, Jashore, Bangladesh; 7 Department of Biochemistry and Molecular Biology, Bangabandhu Sheikh Mujibur Rahman Science and Technology University, Gopalganj, Bangladesh; 8 Fermentation Engineering Major, School of Biotechnology, Jiangnan University, Wuxi, PR China; 9 Immunoinformatics and Vaccinomics Research Unit, RPG Interface Lab, Jashore, Bangladesh

**Keywords:** Phytochemical, isovitexin, COVID-19, molecular docking, dynamic simulation, hACE2 receptor, prophylactic agent, target specificity

## Abstract

**Objective:**

Despite the development of several vaccines against severe acute respiratory syndrome coronavirus-2, the need for an additional prophylactic agent is evident. In recent *in silico* studies, isovitexin exhibited a higher binding affinity against the human angiotensin converting-enzyme 2 (hACE2) receptor than existing antiviral drugs. The research aimed to find out the point specificity of isovitexin for the hACE2 receptor and to assess its therapeutic potential, depending on the stability of the isovitexin–hACE2 complex.

**Materials and Methods:**

The pharmacokinetic profile of isovitexin was analyzed. The crystal structure of the hACE2 receptor and the ligand isovitexin were docked to form a ligand–protein complex following molecular optimization. To determine the isovitexin–hACE2 complex stability, their binding affinity, hydrogen bonding, and hydrophobic interactions were studied. Lastly, the root mean square deviation (RMSD), root mean square fluctuation, solvent accessible surface area, molecular surface area, radius of gyration (Rg), polar surface area, and principal component analysis values were found by simulating the complex with molecular dynamic (MD).

**Results:**

The predicted Lethal dose_50_ for isovitexin was 2.56 mol/kg, with an acceptable maximum tolerated dose and no hepatotoxicity or AMES toxicity. Interactions with the amino acid residues Thr371, Asp367, Glu406, Pro346, His345, Phe274, Tyr515, Glu375, Thr347, Glu402, and His374 of the hACE2 protein were required for the high binding affinity and specificity of isovitexin. Based on what was learned from the MD simulation, the hACE2 receptor-blocking properties of isovitexin were looked at.

**Conclusions:**

Isovitexin is a phytochemical with a reasonable bioactivity and safety profile for use in humans, and it can potentially be used as a hACE2-specific therapeutic to inhibit COVID-19 infection.

## Introduction

The coronavirus disease 2019 (COVID-19) caused by the severe acute respiratory syndrome coronavirus-2 has been identified as the solely responsible pathogen for the global pandemic, which is progressively self-mutated and highly transmissible. Respiratory symptoms such as cough, fever, and shortness of breath characterize this illness. Most often, the virus can cause pneumonia, kidney failure, severe acute respiratory syndrome, and lung failure [1], resulting in 4.55 million deaths globally [2]. The interaction between host cells and SAR-CoV-2 dictates the extent of cell infection and replication and disease progression. The human angiotensin converting-enzyme 2 (hACE2) receptor plays a pivotal role in COVID-19’s entry. Myocardial cells, endothelial cells, esophageal cells, artery smooth muscle cells, bladder urothelial cells, and other tissues express the ACE2 receptor [3]. The ACE2 proteins are found in greater abundance in the lungs [3] and kidneys [4], which reveals the pneumocytes’ sensitivity to COVID-19 [5]. Among the several possible manifestations of severe acute respiratory syndrome coronavirus-2 (SARS-CoV-2), one notable manifestation is the neurological consequences. It is believed to be a complex mechanism and is associated with symptoms such as ischemic stroke, pyramidal signs, hypo/anosmia, delirium, and impaired consciousness [6]. However, it should be noted that the neurological consequences of SARS-CoV-2 may be secondary to the manifestations of hypoxia and ARDS [7]. It has already been reported that hypoxia and ARDS can be the neurological disorders of COVID-19 progression [8].

To invade the host, COVID-19 uses S1 and S2 glycoproteins. S1 is further cleaved by host proteases that enable its attachment to the peptide domain of the ACE2 receptor [9]. This binding further requires cleavage of ACE2 at the ectodomain region, facilitated by ADAM17 protein [10], and cleavage by TMPRSS2 [11]. These enable effective viral entry into the cells. The S1 protein of SARS-CoV-2 selectively binds to ACE2 (from ACE1) via the receptor-binding domain (RBD). [12]. The RBD of SARS-CoV-2 has nearly 20 times higher affinity for ACE2 receptors than the RBD of SARS-CoV.

Plants and herbs have been an essential source of medicine since ancient times. Medicinal plants contain phytochemicals that have therapeutic properties to help treat prevalent diseases such as malaria, tuberculosis, diarrhea, and asthma [13]. Bioactive components from plant sources have received much attention in pharmaceutical sciences to develop antiviral treatments for tropical diseases like dengue, chikungunya, yellow fever, and AIDS [14]. Upon the outbreak of the coronavirus, 200 bioactive compounds from Chinese medicinal plants were tested for treatment against SARS-CoV-2. Some of the herbal extracts from medicinal plants have shown the capacity to inhibit SARS-CoV replication [15]. Hypericin, an herbal compound from *Hypericum perforatum*, has a high affinity for ACE2 receptors. However, laboratory studies of this compound are still required [16]. Isovitexin is a plant-derived bioactive compound [17], which has been used as a traditional Chinese medicine for curing diseases as it contains multivalent efficacy in curing diseases [18]. Isovitexin shows antioxidant, proinflammatory, and anti-inflammatory effects as reported [19,20].

Taking all of these things into account, the goal of this research was to study the point specificity of isovitexin to the hACE2 receptor by using molecular docking and molecular dynamic simulation to find out how isovitexin could be used as a prognostic drug against COVID-19 infection. 

## Materials and Methods

### Optimization of the hACE2 receptor as a macromolecule

The resolved crystal structure of the human ACE2 receptor (1R4L) was obtained from the PDB [21]. As part of the molecular optimization all water molecules, ions, nonstandard amino acids, heteroatoms, and extra chain subunits apart from the A chain were removed using the UCSF Chimera software (version 1.15). The protein energy was minimized by the YASARA Dynamics. Afterward, the optimized protein structure was saved as a .pdb file.

### Prediction of active site amino acids of the protein

**Figure 1.**
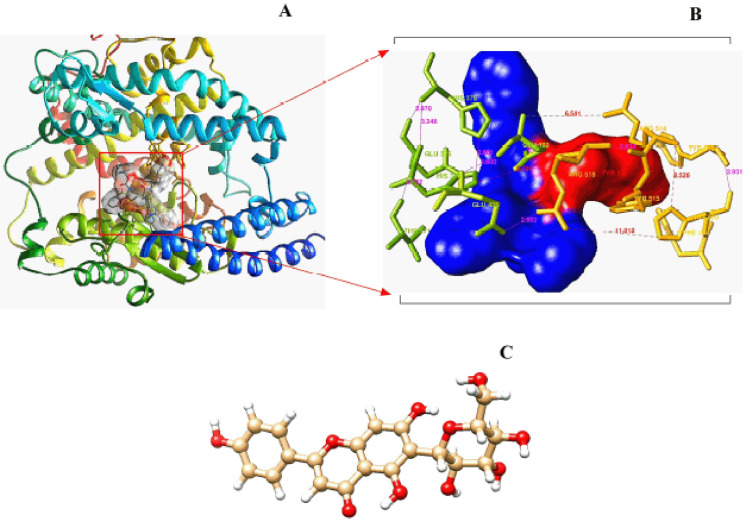
Illustration of the receptor macromolecule (A) with its best active site for super docking (B) and optimized crystal structure of the ligand isovitexin (C).

In the quantitative observation, eight binding pockets were estimated for the hACE2 receptor protein, obtained from the COACH-D algorithm [22,23]. After evaluating the predicted binding energy and involvement of particular residues, the top binding pockets were chosen from the eight found (Fig. 1A). The COACH-D (https://yanglab.nankai.edu.cn/COACH-D/) algorithm was used to analyze the molecular super-docking point [23] of the optimized hACE2, indicating the best active site by monitoring its binding affinity (Fig. 1B) and the neighboring amino acids at the predicted super-docking pose. 

### Pharmacokinetic profiling of isovitexin

The pharmacokinetic properties of isovitexin following its absorption, distribution, metabolism, elimination, and toxicity (ADMET) and quantitative structure–activity relationship (QSAR) profiles were studied [24]. The parameters that followed Lipinski’s rule were initially evaluated using Swiss ADME and Molinspiration Cheminformatics [22]. Finally, the toxicity level was analyzed using the pkCSM server system [23]. The QSAR profile was determined using admetSAR 2.0 preliminary. On the PASS server [24], a secondary validation of the QSAR assessment of isovitexin was carried out to look at its antimicrobial and anti-infective properties. 

### Optimization of isovitexin as the ligand

The structure of isovitexin (PubChem CID: 162350) was obtained from the PubChem database [25]. The energy minimization was conducted by UCSF Chimera 1.14 [26]. After minimization, the compound was converted into the “mol2” format to perform molecular docking (Fig. 1C).

### Molecular docking

Molecular super docking on the best binding active site was carried out using the PyRx 0.8 software package (https://pyrx.sourceforge.io/downloads) which functions on the AutoDock Vina interface [27]. After obtaining the results from the docking, the values of root mean square deviation (RMSD) (Å) and binding energy were conserved as .csv files [27]. 

### Visualization of isovitexin–hACE2 receptor complex

After completing the docking of ligands on the super active binding site of the macromolecule, the “pdbqt” files were redeemed from the PyRx tool. The complex formed between the protein and ligand was visualized via the DS Visualizer (ver. 3.0) and PyMOL (ver. 2.4.1). The data obtained from PyMOL were converted into .pdb format. LigPlot (version 2.2) [28] was used to look at how the docked complexes interacted with each other in polar and hydrophobic ways. 

### Molecular dynamic simulation (MDS)

Initially, the hACE2 (ligand-free) optimized macromolecule was simulated for 10 ns using CABS-flex 2.0 [29]. LARMD was operated to determine the B-factor, solvent accessible surface area (SASA), root mean square fluctuation (RMSF), RMSD, and principal component analysis (PCA) values after MD simulation for 3.1 ns of the isovitexin–hACE2 complex [30]. Afterward, the Desmond–Schrodinger software was run for 20 ns to figure out the molecular surface area (MolSA) (Å^2^), H-bonds, radius of gyration (Rg) (nm), RMSD (Å), polar surface area (PSA) (Å^2^), SASA (Å^2^), and RMSF (Å) values of the drug–receptor complex [31]. 

### Statistical analysis and graphical representation

The resultant values of the molecular dynamic simulation were statistically analyzed using GraphPad Prism software package (ver. 8.0.1) [32–34] and R Studio (ver. 4.0.2) [35–37]. 

## Results

### Pharmacokinetic analysis of the ligand

Based on the ADMET analysis, the ligand isovitexin violated one of the rules of Lipinski. The intestinal absorption of the ligand was more than 60%. The ligand demonstrated a high excretion rate and had an acceptable maximum tolerated dose (MTD) for human consumption. The value of log P was 0.09 and the lethal dose (LD)_50_ was 2.56 mol/kg. Isovitexin did not show hepatotoxicity or AMES toxicity.

### Identification of the protein’s active site

The molecular optimization reduced the energy from −52,407.7 to −113,291.2 kJ/mol for optimized hACE2 (Fig. 1A). The “COACH-D” algorithm predicted that the active site residue positions of hACE2 receptors (273, 345, 346, 347, 348, 367, 371, 374, 375, 378, 382, 401, 402, 449, 503, 504, 505, 510, 512, 515, and 519) (Fig. 1B) were critical for ligand attachment. Based on COACH-D predictions, the binding can be regarded as effective while yielding a binding energy of −6.9 kcal/mol. 

### Postmolecular super-docking analysis

**Figure 2.**
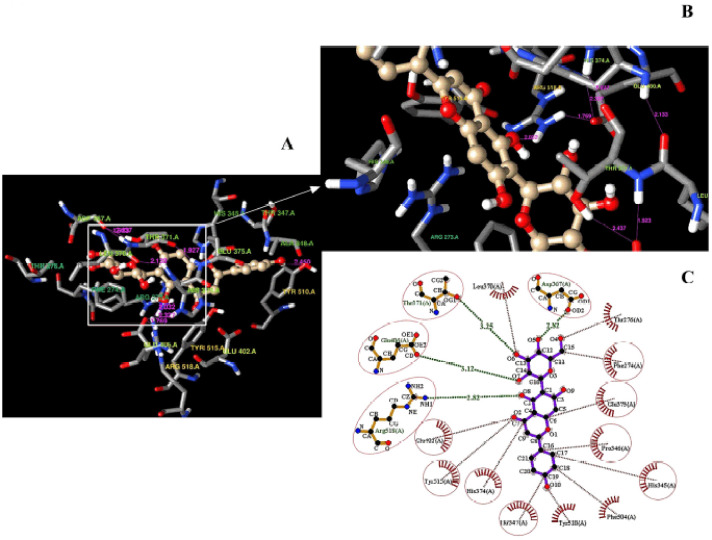
Demonstration of the super docking of isovitexin inside the hACE2 receptor (A); the amino acid residues involved in the supra-molecular docking position (B); the formation of hydrogen bonds and non-covalent (hydrophobic) interactions into the docked complex (C). The hydrogen bonds are indicated using the pink lines (A and B) as 3D confirmation. The most stable hydrogen bonds (green line) and hydrophobic interactions (red lines) are shown as 2D confirmation (C).

Isovitexin displayed a compact interaction with Glu406 (3.12 Å), Arg (2.82 Å), Asp367 (2.82 Å), and, Thr371 (3.25 Å), and also with hydrophobic interactions as Tyr515, Glu402, Phe274, Pro346, Thr347, His345, Glu375, and His374 (Fig. 2A and B). The hydrogen bonds formed between isovitexin and hACE2 have been marked with green lines indicating the atomic distances (Å), whereas the hydrophobic interactions are red (Fig. 2C). 

### Molecular dynamic simulation (20 ns)

**Figure 3.**
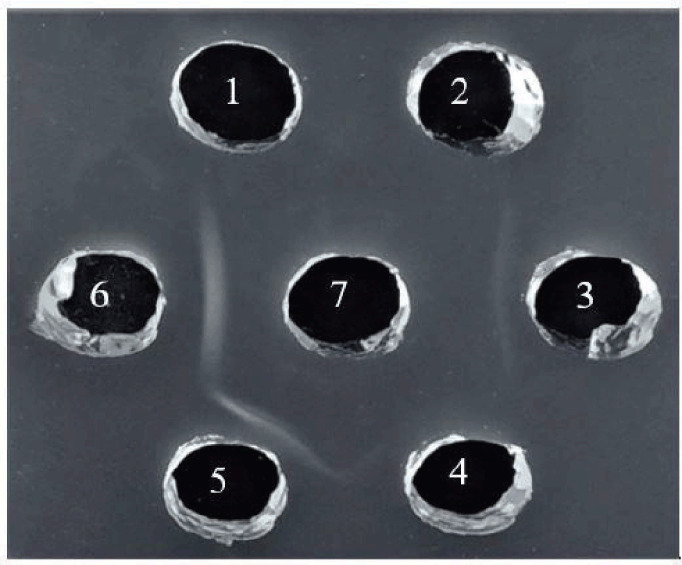
Illustration of the isovitexin–hACE2 receptor complex profile at the 3.1 ns of MDS (A); a clustering dendrogram derived from the principal component analysis (B); a heat map of the molecular dynamics residual cross-correlation matrix (C); PCA cluster analysis with trajectory frames from blue to red in order of time (D), which are recovered by changing conformations from black to red (E); PCA scree plot showing the proportion of variance against its eigenvalue rank, where the first three eigenvectors contributes over 40% of the total variance (D and E).

The interactions among different atoms of isovitexin and circulating eight amino acid residues have been studied at the point of 3.1 ns of the MDS (Fig. 3A). The distance between Tyr510 and isovitexin was observed to be 2.450 Å; Thr371 and Asp367 was 2.437 Å; Asp367 and Leu370 was 1.923 Å; and Leu370 and His374 was 2.133 Å. The bond length between His374 and Arg319 was 1.927 Å; Arg319 and Glu409 was 1.769 Å; Glu406 and His374 was 2.327 Å; and finally, Tyr510 and isovitexin was 2.023 Å. The cladogram represents the group of amino acid residues involved in the point of ligand complexing during the dynamic simulation based on the hypothetical relationship among them at the point of 3.1 ns (Fig. 3B). The residue cross-correlation map was generated (Fig. 3C) depending on the principal component analysis of the hACE2 receptor resulting from the MDS. The red spots indicate regions where residues have correlated with each other, and the blue spot indicates anti-correlation in the complex. The residues from −0.2 to −1 showed anti-correlation, whereas the residues from 0.2 to 1 showed correlation. This indicates a 50:50 correlated and anti-correlated residue ratio in the docked structure (Fig. 3D). The two clusters of the frame based on the first three eigenvectors (PCs) represent the active and inactive states of the protein. The trajectory shown in Figure 3E recovered from the trajectory frames in Figure 3D is divided into two different clusters of black and red through the top three PC spaces. The eigenvector 1 (PC1) accounts for about one-fourth of the overall variance (25.06%) which strongly dominates the overall variance, while PC2 (10.45%) and PC3 (5.76%) contributed to the remaining variance. The first three components together make up 41.3% of the total variance. The rest of the individual component contributions dropped below 4.3%.

**Figure 4.**
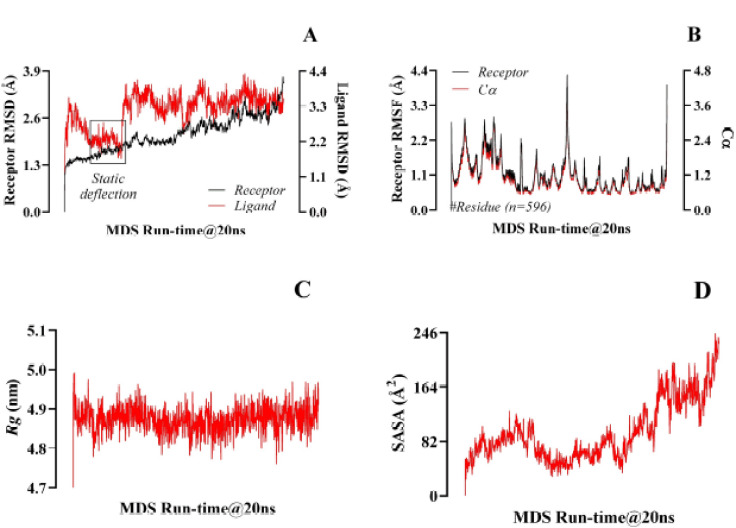
Representation of the threshold spectrum of RMSD (A), RMSF with Cα (B), Rg (C), and SASA (D), resulting from the molecular dynamic simulation of 20 ns runtime

Although the ligand and the receptor went through conformational fluctuations at different RMSD levels before the first half of the simulation time, they equilibrated at the beginning and during the final half of the simulation time. The highest RMSD value displayed by isovitexin ranged between 1.98 Ǻ and 3.752 Ǻ (Fig. 4A). The fluctuations of RMSF values were within a limited range for most of the simulation period, meaning 0.96 Ǻ and 4.3 Ǻ (Fig. 4B). The Rg values resulted in a 4.76–4.98 nm spectrum and showed prominent efficiency (Fig. 4C). The SASA analyzed all the hACE2 residues (597AA), which were associated with the ligand’s strength. Although there were considerable fluctuations in SASA values (57.8 Ǻ2–244.8 Ǻ2), they remained within a narrow range for most of the simulation time (Fig. 4D).

**Figure 5.**
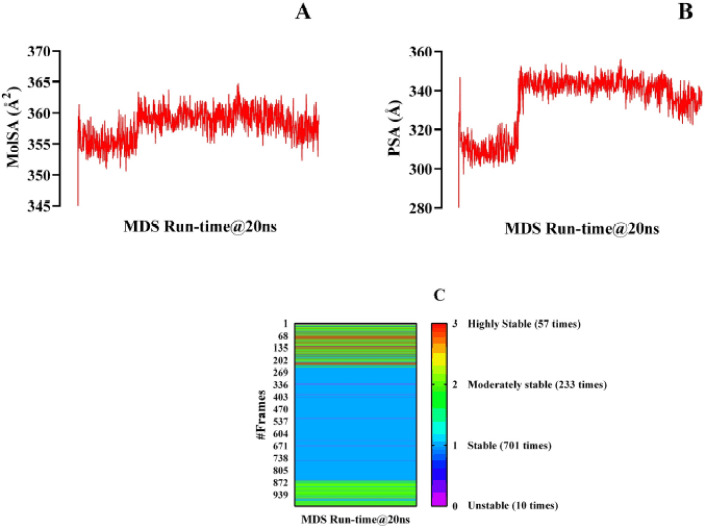
Illustration of the MolSA (A), PSA (B), and the number of intramolecular hydrogen bonds per frame during the 20 ns runtime of the molecular dynamic simulation considering its 1001 different frames (C).

To determine the MolSA, a probe of radius 1.4 Å was set, which is similar to a water molecule’s van der Waals surface area. During the 20 ns simulation period, isovitexin showed a maximum MolSA value of 364.806 Å2 and did not exhibit much fluctuation (Fig. 5A). The result is acceptable since the MolSA value did not fluctuate much. The PSA value refers to the polar area present in isovitexin and 1R4L proteins, which has a better correlation with the H-bonds and also indicates lipophilicity. The PSA presented here is the summation of all the surface contributions of the polar fragments present in the docked complex (Fig. 5B). The figure shows that isovitexin has a wide range of fluctuations in PSA from 297.167Å to 356.201Å. For the 20 ns MDS course, 1001 frames of trajectories were extracted. The isovitexin–hACE2 complex exhibited 3 intramolecular hydrogen bonds in 57 frames, rendering the complex highly stable. The presence of 2 hydrogen bonds in 233 frames and 1 hydrogen bond in 701 frames ensures the strength of the ligand–protein complex. There were only 10 frames with no hydrogen bond formation out of the 1,001 frames (Fig. 5C).

## Discussion

COVID-19 radically impacted lives on a global scale. In particular, developed countries with highly efficient medical infrastructure have been suffering from significant death rates [38]. Multiple vaccines have been developed to equip the public with long-lasting immunity to battle infectious diseases [39]. The developing, and underdeveloping countries of Africa, Asia, and Latin America are the worst sufferers [40]. Vaccine shortages in developing and poor countries are caused by a lack of healthcare infrastructure and long-term vaccine conservators. The other obstacle to the vaccination drives in developing countries is the hesitance of people to vaccinate. To ameliorate such problems, selecting therapeutics like phytochemicals or natural compounds in the form of oral tablets or solutions could be another possible step. *In silico* studies can help find these potential preventive drugs for COVID-19. They can be used to help protect people who do not want to get vaccinated from the virus and end the pandemic sooner.

According to ADMET profiles, as evidenced by a log *p* value (0.09), isovitexin exhibited low lipophilicity. This indicates a possibility of poor membrane permeability and high renal clearance of unmetabolized parents [41]. As predicted by the pkCSM web interface, isovitexin may have a strong rate of excretion with around 60% of absorption in the intestine, in agreement with the log P value. The LD_50_ score was within an acceptable range, when the hepatotoxicity and AMES toxicity predictions were also returned with negative results, thus indicating that the ligand is safe. Also, the MTD value for isovitexin showed that it can be given to humans safely because the tolerance stayed within a safe range.

A good number of intramolecular interactions were evident in the docked complex structure (Fig. 2A–C; 3A). The presence of such intramolecular interactions results in favorable protein–ligand orientation, leading to high binding affinity [42]. The high number of intramolecular interactions between the protein and ligand stabilizes the geometry of two interacting molecules [43]. A clustering dendrogram has been used based on PC1, PC2, and PC3 to identify the parental linkages of the active chemicals (Fig. 3B). The correlation matrices, in the form of heat maps, portray the residual interactions of the docked complexes [44]. A heat map provides visual insight into the association between molecules and their coefficient distances [45]. Here, smart correlations were notified between isovitexin and hACE2 (Fig. 3C) in a manner of anti-correlated and correlated residues with 50:50 chances.

To identify the protein–ligand stability, MDS was utilized. Eight different parameters (i.e., intramolecular H-bonds, Rg, MolSA, RMSD, SASA, RMSF, PCA, and PSA) were assessed up to a 20-ns simulation period. To reduce the complexity of collective motions from MD simulation trajectories, PCA was run to assess the collective dynamic behavior of the protein–ligand complex [46]. PCA also provides information on conformational differences in molecular dynamics trajectories [47]. Clustering structures enable a comprehensive assessment of the relationship between individual structures in light of the major conformational changes observed [48]. Clustering along PC1 and PC2 (Fig. 3D and E) describes how these conformational changes render the structural relativity during the simulation periods. The high and low clusters demonstrate the significant and nonsignificant stabilities of complexes, respectively [49]. In this research, two clusters comprising blue-to-red spots in response to the passage of time have been generated, referring to the increased stability with the structural alterations in hACE2 following the ligand’s attachment (Fig. 3D).

The RMSD value indicates the average shift in the position of atoms (or a group of atoms) when comparing one frame of atomic orientation with another [50]. This parameter allows a comparison between different chemical structures and helps to reduce a large number of predicted conformations to a manageable number [51]. The larger the RMSD value, the more it indicates a less stable docked complex over the simulation time [52]. In this study, the ligand and the receptor had a harmonic structural change for most of the simulation time, maintaining an overall similar pattern of trajectory until the end of the simulation time (Fig. 4A). This indicates a stable complex between the ligand and the receptor. The RMSF describes the fluctuation of atomic position where the residual fluctuations are estimated with the status of the Cα values. In this study, over a 20 ns runtime, the RMSF values were estimated and the fluctuation range was found to be very satisfactory (Fig. 4B). In any *in silico* study, the upper and lower ranges represent the inferior and vigor complex stabilities, respectively [51,52].

The Rg value helps in reexamining the structural activity of proteins. The Rg values can be negatively reinforced with the protein’s fold changes [53], considering that Rg is a very flexible way of determining the drug–protein stability. The range remained within the positive Rg trajectories in this research (Fig. 4C).

SASA helps to assess the interaction between water molecules and the surface of the docked complex [54]. A complex with high SASA values indicates high solvent accessibility, leading to increased instability. On the contrary, a complex with a low SASA value indicates that it is relatively stable [27]. In the current study, despite significant fluctuations in SASA values, they remained within an acceptable range (Fig. 4D). This indicates that the isovitexin–hACE2 complex is fairly stable.

MolSA is also linked to the stability of the protein–ligand complex [55]. A high MolSA value indicates an unstable protein–ligand complex, whereas a lower MolSA value indicates a comparatively stable complex [56]. In this study, the MolSA was primarily stable and did not show any remarkable fluctuations (Fig. 5A). Thus, the isovitexin–hACE2 complex can be considered stable. PSA is a representative of the blood–brain barrier status of drugs [57]. If the PSA of a drug is more than 40 or less than 90Å, it may cross the blood–brain barrier. In the current research, PSA ranged between 297.167 Å^2^ and 356.201 Å2 referring to isovitexin’s failure to trans-pass the BBB (Fig. 5B). Since the neurological problems caused by COVID-19 might not happen if ARDS and hypoxia do not happen, there is no need for a preventative drug to cross the blood–brain barrier.

Hydrogen bonding accounts for the final profiles of the drug–receptor complexes at the very end point of the MD simulation [58]. The more H-bonds formed, the more significant the complex [59]. The adequacy of hydrogen bond regeneration and the drug–receptor complex have been identified quite vigilantly. In over 99% of the simulation time, one or more hydrogen bonds were present, which suggests that the isovitexin–hACE2 complex is highly stable (Fig 5C). In recent times, to avoid drug resistance to the antiviral drugs, a range of bacteriocins (antimicrobial proteins) have been emphasized to combat SARS-CoV-2 [60], isolated from different probiotic microorganisms [61]. It is suggested that a secondary immune response successfully takes place following a proper opsonization process [62]. 

## Limitations

One of the primary limitations of pharmacophore-based drug design is the sheer complexity of molecular dynamics. The analysis duration ranges from tens of nanoseconds to microseconds. The approach is computationally demanding and depends on the scale of the simulated systems. In this study, molecular dynamics simulation was run for 20 ns, but a higher runtime would have been more informative. A very small chemical space has been sampled by working with only one molecule. Thus, the next step would be to perform a substructure search with the SMILES notation, find other similar phytochemicals, and perform similar assessments with a more extensive chemical set. However, this was out of the scope of this study. Additionally, there was a gap in the N-terminal of the protein crystal structure used for the docking simulations. The gap was not filled as the COACH-D top binding site predictions with the homology model did not suggest any important amino acid residue at the N terminus for ligand binding. Thus, this study could not address any topological errors arising from that gap region during molecular dynamics simulation.

## Conclusion 

In the present study, the potential of isovitexin as an ACE2-selective prophylactic agent against COVID-19 was evaluated from an *in silico* perspective. The pharmacokinetic properties of isovitexin were evaluated using ADMET and QSAR profiling. The protein–ligand interactions were analyzed after molecular docking simulation with the optimized ligand and macromolecule. Finally, molecular dynamics simulation was conducted to assess PCA, RMSD, RMSF, Rg, SASA, MolSA, and PSA and intra-H bonds. The molecular docking analysis revealed a favorable binding affinity of the ligand for the human ACE2 receptor. The post-docking assessment in LigPlot+ revealed interactions with the amino acid residues (Glu406, Thr371, Asp367, His345, Pro346, Phe274, Glu375, Tyr515, Thr347, His374, Arg518, and Glu402) were important for optimal binding affinity and isovitexin–hACE2 complex stability. PCA analysis demonstrated a significant conformational change in hACE2 caused by binding to isovitexin and increased stability over time. Further molecular dynamics simulation studies revealed that isovitexin possesses good stability and dynamics within the hACE2 active site. Also, more *in vitro* and in vivo tests can be carried out to ure out the pharmacodynamic profile of isovitexin with COVID-19’s protective effect.

## List of abbreviations

ARDS, Acute respiratory distress syndrome; PDB, Protein data bank; UCSF, University of California, San Francisco; SARS-CoV-2: Severe acute respiratory syndrome coronavirus-2; hACE-2: Human angiotensin converting-enzyme 2; MTD: Maximum tolerated dose; LD: Lethal dose; RMSD: Root mean square deviation; RMSF: Root mean standard deviation; SASA: Solvent accessible surface area; MolSA: Molecular surface area; Rg: Radius of gyration; PSA: Polar surface area; PCA: Principal component analysis; ADMET: Absorption, distribution, metabolism, elimination, and toxicity; QSAR: Quantitative structure–activity relationship; MDS: Molecular dynamic simulation
